# Identification of the 12q24 locus associated with fish intake frequency by genome-wide meta-analysis in Japanese populations

**DOI:** 10.1186/s12263-019-0646-6

**Published:** 2019-07-05

**Authors:** Maki Igarashi, Shun Nogawa, Kaoru Kawafune, Tsuyoshi Hachiya, Shoko Takahashi, Huijuan Jia, Kenji Saito, Hisanori Kato

**Affiliations:** 1 Laboratory of Health Nutrition, Department of Applied Biological Chemistry, Graduate School of Agricultural and Life Sciences, The University of Tokyo, 1-1-1, Yayoi, Bunkyo-ku, Tokyo, 113-8657 Japan; 2 Department of Molecular Endocrinology, National Research Institute for Child Health and Development, 2-10-1 Okura, Setagaya-ku, Tokyo, 157-8535 Japan; 3 Genequest Inc, 5-29-11 Siba, Minato-ku, Tokyo, 108-0014 Japan; 4 Genome Analytics Japan Inc, 15-1-3205, Tomihisa-cho, Shinjuku-ku, Tokyo, 162-0067 Japan

**Keywords:** Genome-wide association study, Single nucleotide polymorphism, Fish intake frequency, 12q24, Alcohol drinking

## Abstract

**Background:**

Japan is traditionally a country with one of the highest levels of fish consumption worldwide, although the westernization of the Japanese diet has resulted in the reduction of fish consumption. A recent meta-analysis of genome-wide association studies (GWASs) on Western populations has identified a single nucleotide polymorphism (SNP) associated with fish intake frequency. Here, we examined the genetic basis for fish intake frequency among Japanese individuals.

**Results:**

We conducted a meta-analysis of a GWAS including 12,603 Japanese individuals and identified a susceptibility locus for fish intake frequency at 12q24 (lead variant was rs11066015, *P* = 5.4 × 10^−11^). rs11066015 was in a strong linkage disequilibrium with rs671, a well-known SNP related to alcohol metabolism. When adjusted for alcohol drinking, the association between rs11066015 and fish intake frequency was substantially attenuated. Subgroup analysis revealed that the effect of the 12q24 variant on fish intake frequency was stronger in males than in females (*P* for interaction = 0.007) and stronger in the older subgroup than in the younger subgroup (*P* for interaction = 0.006).

**Conclusions:**

Our findings suggest that the 12q24 locus is associated with fish intake frequency via alcohol drinking. This study can help contribute to personalized nutrition information, suggesting that fish intake should be promoted to consumers who have the rs11066015 minor allele, which is genetically linked to low fish intake frequency, especially in male and older individuals.

**Electronic supplementary material:**

The online version of this article (10.1186/s12263-019-0646-6) contains supplementary material, which is available to authorized users.

## Background

Traditionally, Japan consumes large amounts of fish. On the other hand, in recent years, the consumption of meat has surpassed the consumption of fish and seafood, as dietary habits are becoming increasingly westernized [1]. Omega-3 fatty acids, such as eicosapentaenoic acid (EPA) and docosahexaenoic acid (DHA), which are abundant in blueback fish, have been reported to prevent lifestyle-related diseases and to improve symptoms of bipolar disorder and hyperactivity by lowering blood low-density lipoprotein cholesterol and triglycerides and suppressing serotonin levels [2, 3]. Thus, the importance of consuming fish is becoming globally recognized owing to these health benefits.

Mozaffarian et al. conducted a meta-analysis of genome-wide association studies (GWASs), including 17 Western cohort studies, and reported associations between rs9502823 (*β* = − 0.029, *P* = 2.0 × 10^−8^) and fish intake frequency [4]. The SNP rs9502823 is located on chromosome 6 near *AX747250*, which produces a non-protein-coding transcript. Moreover, whereas rs9502823 is in complete linkage disequilibrium (LD) with rs72838923, which is near *LOC285768*, the function of this gene is also unknown. Therefore, the mechanism by which rs9502823 is associated with fish consumption has not yet been clarified. Hitherto, there have been no previous reports on GWASs that have examined fish intake frequency in East Asian populations.

We carried out a meta-analysis of a GWAS that examined the fish intake frequency in 12,603 participants recruited from eight residential regions in Japan. We identified a single locus associated with fish intake frequency and performed a genetic epidemiological analysis, including subgroup analysis, on the identified locus.

## Results

### Genome-wide meta-analyses in Japanese populations

The characteristics of the study subjects according to eight regional groups from a previous study [5] are shown in Table 1. The fraction of females ranged from 45.1% in Kanto-Koshinetsu to 51.7% in Hokkaido. The mean age ranged from 48.9 years old in Okinawa to 51.6 in Hokkaido. The fish intake frequency in Hokkaido and Tohoku—northern areas of Japan—was slightly higher than that in other areas (ANOVA, *P* = 0.003).

**Table 1:** Characteristics of the study participants

	Hokkaido	Tohoku	Kanto-Koshinetsu	Tokai-Hokuriku	Kinki	Chugoku-Shikoku	Kyushu	Okinawa	*P*
*N*	480	829	4959	1544	2110	1042	1165	118	–
Female, %	51.7	46.9	45.1	46.9	47.6	47.2	50.0	45.8	0.03
Age, year (mean ± SD)	51.6 ± 13.2	50.7 ± 12.8	49.6 ± 13.0	50.4 ± 13.0	50.5 ± 13.5	51.1 ± 13.3	50.7 ± 13.4	48.9 ± 12.3	< 0.001
BMI, kg/m^2^ (mean ± SD)	23.3 ± 3.6	23.3 ± 3.9	23.1 ± 3.8	22.9 ± 3.6	22.9 ± 3.6	23.1 ± 3.7	23.1 ± 3.7	23.9 ± 4.0	0.001
Fish intake frequency, per week (mean ± SD)	2.3 ± 2.0	2.4 ± 1.9	2.1 ± 1.9	2.2 ± 1.9	2.1 ± 1.7	2.1 ± 1.8	2.0 ± 1.8	1.9 ± 1.6	0.003
Current alcohol drinkers, %	60.1	63.7	64.3	56.2	60.7	58.3	60.5	70.3	< 0.001
Current alcohol consumption, g/day (mean ± SD)*	12.5 ± 14.5	11.8 ± 13.3	12.2 ± 14.3	10.5 ± 12.1	10.6 ± 12.2	10.4 ± 11.7	11.9 ± 14.1	14.0 ± 16.3	< 0.001
Inflation factor	1.015	0.999	0.993	1.020	1.005	1.018	1.001	1.009	–

*BMI* body mass index, *SD* standard deviation

*P* values were calculated using *χ*^2^ test for sex and current alcohol drinkers or ANOVA for other variables

*Among current drinkers

**Fig. 1:**
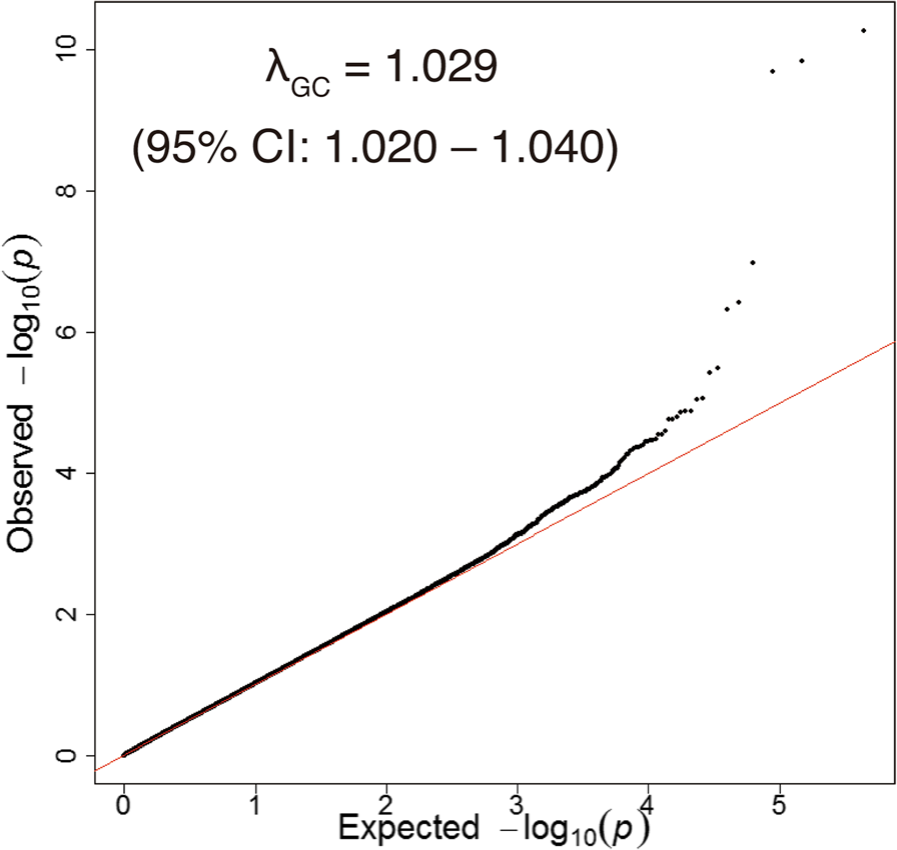
*P* value distribution in the genome-wide meta-analysis. A quantile-quantile plot for the genome-wide meta-analysis is shown. The *x*-axis represents theoretical − log_10_ *P* values, and the *y*-axis represents observed − log_10_ *P* values. The red line indicates *y* = *x*

Regional group-specific genome-wide scans, which adjusted for age, sex, and population stratification, yielded inflation factors (*λ*) that ranged from 0.993 to 1.020 (Table 1). The meta-analysis generated inflation factors of 1.029 (95% confidence interval [CI] 1.020–1.040; Fig. 1), indicating that a minimal level of inflation was observed.

**Fig. 2:**
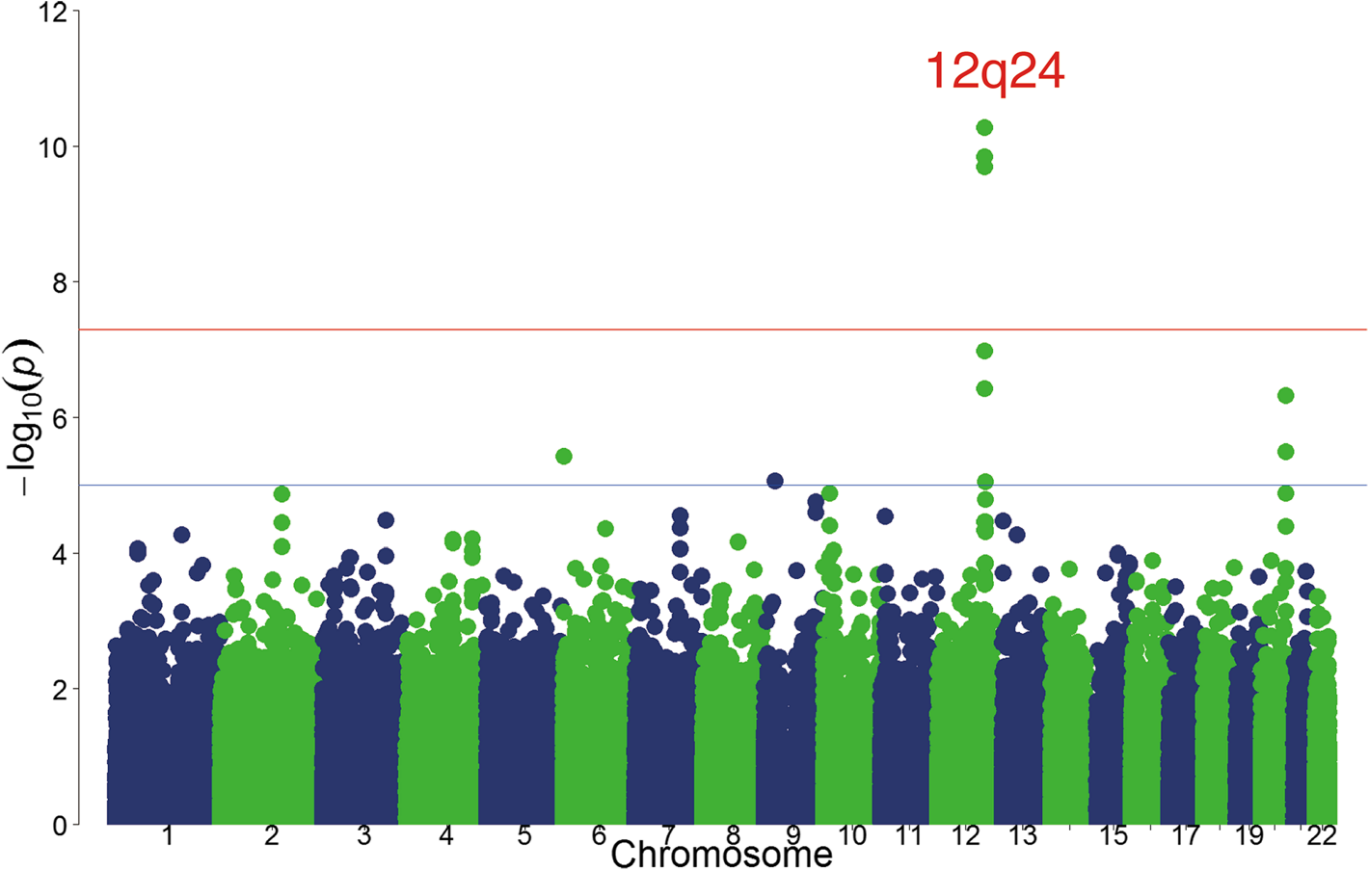
Genome-wide meta-analysis of fish intake frequency. The *x*-axis represents chromosomal positions, and the *y*-axis represents − log_10_ *P* values. The red and blue horizontal lines indicate the genome-wide significance (*P* = 5 × 10^−8^) and suggestive significance (*P* = 5 × 10^−5^) levels, respectively

The genome-wide meta-analysis identified four candidate loci that achieved suggestive significance (*P* < 1 × 10^−5^), of which 1 (rs11066015) reached genome-wide significance (GWS) (*P* < 5 × 10^−8^) (Fig. 2; Table 2). No evidence of heterogeneity among study regions was observed for rs11066015 (Table 2 and Additional file 1: Table S1). As expected theoretically, the standard error of the regression coefficients decreased as the number of subjects in the region increased (Table 1 and Additional file 1: Table S1), resulting in an evident association between rs11066015 and fish intake frequency in the study areas with large sample sizes (Additional file 1: Table S1). Very similar results of the regional group-specific genome-wide scans were obtained when additionally adjusted for body mass index (Additional file 1: Table S2).

**Table 2:** Lead variants associated with fish intake frequency

SNP	Chr	Position	Gene(s)	EA	NEA	EAF	Beta	SE (beta)	*P*_association_	*I* ^2^	*P*_heterogeneity_
rs11758482	6	2,438,231	*GMDS–C6orf195*	G	A	0.405	0.110	0.024	3.8 × 10^−6^	49.8	0.05
rs12003047	9	25,527,621	*IZUMO3–TUSC1*	C	T	0.015	0.431	0.097	8.7 × 10^−6^	53.3	0.04
rs11066015	12	112,168,009	*ACAD10*	A	G	0.265	− 0.174	0.027	5.4 × 10^−11^	0.0	0.65
rs1201914	20	59,274,641	*CDH26–CDH4*	G	A	0.452	− 0.117	0.023	4.8 × 10^−7^	0.0	0.49

*SNP* single nucleotide polymorphism, *Chr* chromosome, *EA* effect allele, *NEA* non-effect allele, *EAF* effect allele frequency, *SE* standard error

Chromosomal positions are according to the human genome assembly version GRCh37/hg19

### Alcohol drinking, rs11066015, and fish intake frequency

The rs11066015 A allele was linked to the rs671 A allele (*R*^*2*^ = 0.946 in East Asian populations according to the LDlink server [6]), a well-studied variant that is specific to East Asian populations (i.e., monoallelic in other ethnicities) [7, 8] and is associated with a lower level of alcohol drinking [9, 10]. Our data confirmed a strong association between rs11066015 and alcohol consumption levels (*P* = 3.6 × 10^−247^ when adjusted for age, sex, and study region; *P* = 8.7 × 10^−240^ when adjusted for age, sex, study region, and fish intake frequency). To determine whether the association between the 12q24 locus and fish intake frequency was mediated by alcohol drinking, we repeated the association analysis with and without adjustment for alcohol consumption or alcohol intake frequency.

When adjusted for age, sex, and study region, rs11066015 was associated with fish intake frequency (*β* = − 0.174; *P* = 4.3 × 10^−11^) (Additional file 1: Table S3). When additionally adjusted for alcohol consumption, the association was substantially attenuated (*β* = − 0.089; *P* = 1.2 × 10^−3^). In addition, when adjusted for alcohol intake frequency (times per week) instead of alcohol consumption, a similar result was obtained (*β* = − 0.104; *P* = 2.5 × 10^−4^).

### Subgroup analysis stratified by sex and age

We determined whether the effect of rs11066015 on fish intake frequency was heterogeneous among subgroups defined by sex and age. The heterogeneity among subgroups was tested by adding a multiplicative interaction term in the linear regression model adjusted for age, sex, and study region. Subjects were divided into two subgroups––subjects with ages lower than the median age (51 years) were classified into a “younger” subgroup, and subjects with ages equal to or higher than the median age were classified into an “older” subgroup. Alcohol consumption was higher in males than in females and higher in the older group than in the younger group (Additional file 1: Tables S4 and S5). A stronger correlation between fish intake and alcohol drinking frequencies was observed in older males (Additional file 1: Table S6).

**Fig. 3:**
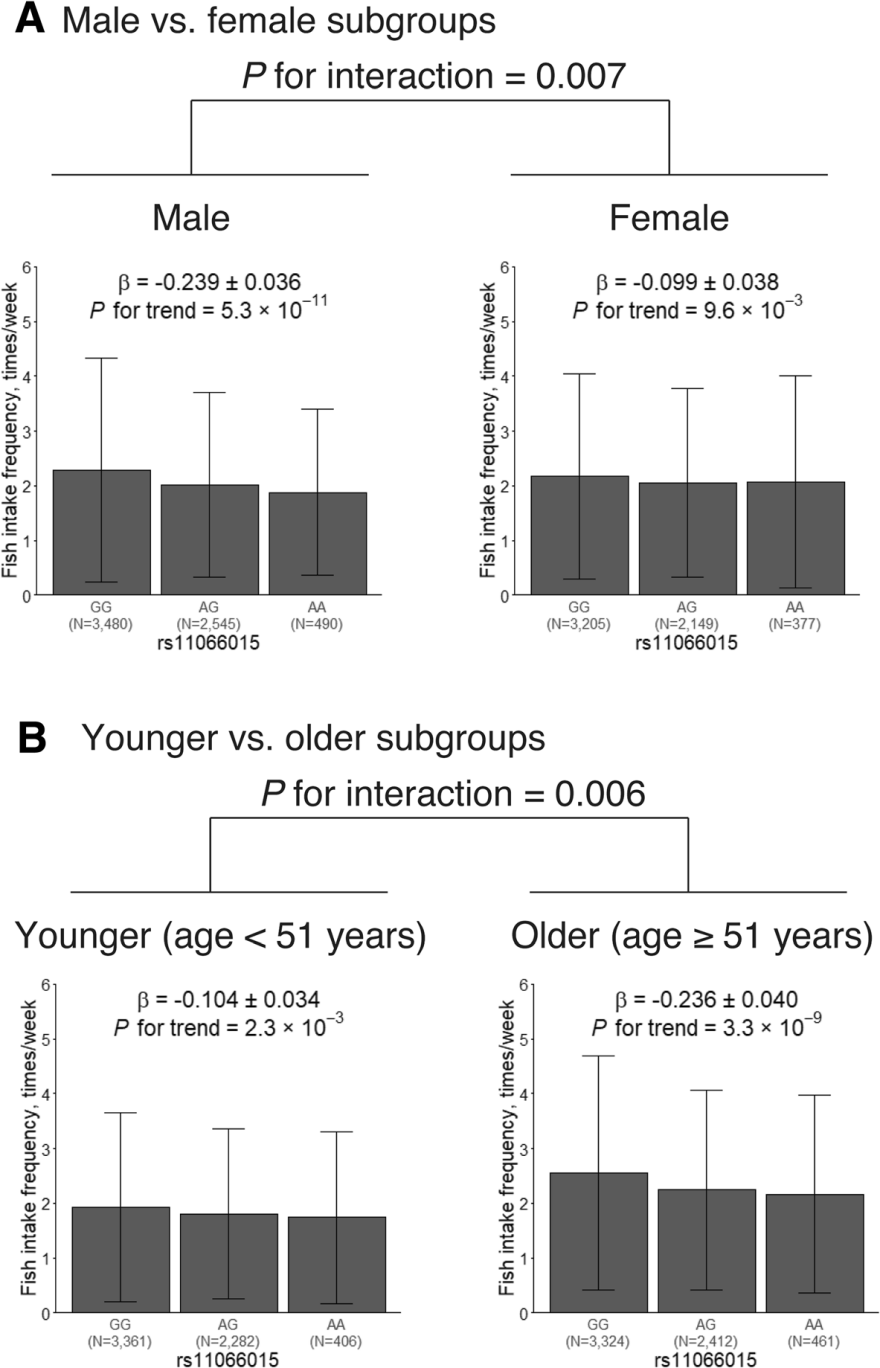
Subgroup analysis stratified by sex and age. The *x*-axis represents the genotype of the lead variant at 12q24 (rs11066015)—i.e., GG, AG, or AA—and the *y*-axis represents fish intake frequency in times per week. **a** Male vs. female subgroups. **b** Younger (age < 51 years) vs. older (age ≥ 51 years) subgroups

The subgroup analysis revealed that the association coefficient in males (*β* = − 0.239; *P* = 5.3 × 10^−11^) was considerably larger than that in females (*β* = − 0.099; *P* = 9.6 × 10^−3^). The effects of the 12q24 locus in males and females were significantly different (*P* for interaction = 0.007; Fig. 3a). This heterogeneity was attenuated when additionally adjusted for alcohol consumption or alcohol intake frequency (Additional file 1: Figures S1 and S2).

The effects of rs11066015 were larger in the older subgroup (*β* = − 0.236; *P* = 3.3 × 10^−9^) than in the younger subgroup (*β* = − 0.104; *P* = 2.3 × 10^−3^). The heterogeneity of the effects among the younger and older subgroups was significant (*P* for interaction = 0.006; Fig. 3b) and was attenuated when additionally adjusted for alcohol consumption or alcohol intake frequency (Additional file 1: Figures S3 and S4).

## Discussion

This study elucidated the association of the 12q24 locus with fish intake frequency. The 12q24 locus is an approximately 2-Mb region with a strong LD, and it has been reported to widely influence metabolic traits [11]. In the 12q24 locus, rs671 is a SNP that is located in the *ALDH2* gene and causes an amino acid substitution [7–10]. The rs671 minor allele reduces the degradation of acetaldehyde, a toxic intermediate metabolite of alcohol, and thus results in reduced alcohol consumption [9, 10]. The association between the 12q24 locus and fish intake frequency was attenuated by adjusting for alcohol consumption or alcohol intake frequency, indicating that the 12q24 locus influences fish intake frequency via drinking habits.

The rs9512823 SNP, previously shown to be associated with fish intake frequency in European and American cohorts [4], is monoallelic in East Asian populations. In contrast, variants (e.g., rs11066015 and rs671) at the 12q24 locus are unique to East Asian populations, including Japanese individuals [7–11]. Thus, it is not surprising that the association between the 12q24 locus and fish intake frequency was not identified in previous studies on populations of European ancestry.

The rs11066015 SNP has a *β* value of − 0.174, indicating that participants’ fish intake decreased by 0.174 times per week, 0.7 times a month, and more than nine times per year for each copy of the minor allele they carried. Generally, portion sizes of fish according to several food frequency questionnaires in Japan range from 60 to 80 g [12, 13]. Hence, it can be estimated that fish consumption decreases by 1.5 to 2.0 g per day for each minor allele carried. However, due to a variety of Japanese dietary habits, this is unlikely to reflect the actual amount consumed. Further studies are necessary to clarify the actual changes in the volume of fish consumed in association with rs11066015, such as obtaining additional information on the details of fish intake in participants or the establishment of food records for fish intake using deep learning.

The association between the 12q24 locus and fish intake frequency was stronger in males than in females and also stronger in the older group than in the younger group. Notably, the same trends were observed in alcohol intake (Additional file 1: Tables S4 and S5), and this trend was reported in the 2012 nutrition survey in Japan [14]. When the association between the 12q24 locus and fish intake frequency was adjusted for alcohol consumption or alcohol intake frequency, the sex and age differences were attenuated (Additional file 1: Figures S1–S4). Accordingly, our results suggested that these differences are mediated by alcohol drinking. That is, older male individuals with drinking habits may be most influenced to 12q24 locus for fish intake frequency.

Another possibility is that the age difference observed in the association between the 12q24 locus and fish intake frequency may be due to a difference in fish consumption between age groups, as fish intake frequency was higher in the older group than in the younger group (Additional file 1: Table S4). Aging-associated changes, such as decreased ability to masticate or digest and lower sensitivity to alcohol because of olfactory decline, are possible factors that influence the increased frequency of fish intake in the older group.

Omega-3 fatty acids produce aldehydes, such as macroaldehyde (MDA), 2-hexenal (HHE), and 4-hydroxy-2-nonenal (HNE), during digestion in the gastric and intestinal lumens [15]. As these aldehydes are degraded by ALHD2, minor allele carriers of rs671 are likely to unconsciously avoid fish intake. In fact, the association *P* values between rs671 and alcohol consumption increased slightly after adjusting for fish intake frequency (from *P =* 3.6 × 10^−247^ to *P* = 8.7 × 10^−240^). Meanwhile, it has been reported that minor allele carriers of rs671 have lower triglyceride levels than homozygous major allele carriers among Chinese drinkers [16]. If the 12q24 locus also affects fish intake in the Chinese population, this report may indicate a suppressive effect of DHA and EPA on triglyceride levels.

In Japan, there is concern about the increased health risks resulting from decreased fish consumption. The results of subgroup analyses suggested that males and older individuals harboring the rs11066015 minor allele had reduced fish intake. Therefore, lifestyle guidance, such as encouraging increased fish intake in these individuals with the rs11066015 minor allele, might result in health-promoting effects. However, further studies, such as GWASs on fish intake in other Asian populations and interventional studies with nutrition education, are needed to clarify whether rs11066015 can be a health-promotion tool.

## Conclusions

In this study, we demonstrated the 12q24 locus has an association with fish intake frequency in the Japanese population. Furthermore, we showed for the first time that there is a genetic linkage between fish intake and alcohol drinking. This study can be expected to contribute to a nutritional improvement in Asian populations, providing a molecular basis for personalized nutrition.

## Methods

### Study subjects

The participants of this study were a part of a direct-to-consumer (DTC) genetic testing service in Japan, “Health Data Lab,” provided by Yahoo! Japan Corporation (Tokyo, Japan) and Genequest Inc. (Tokyo, Japan). The participants were aged ≥ 18 years old and asked to complete internet-based questionnaires covering sociodemographic factors, lifestyle habits, and medical history at the time of enrollment. All participants gave written, informed consent for the general use of their genotype and questionnaire data. After informing the participants of this study’s purpose, an additional agreement was obtained with the opportunity to opt out. Among the 12,621 participants, 1 who opted out was excluded from this study. We obtained approval from the Ethics Committee of Genequest Inc. This study was conducted according to the principles expressed in the Declaration of Helsinki.

### Fish intake frequency

The questionnaires included a question about fish intake frequency, i.e., “How frequently do you eat fish (raw fish, boiled fish, grilled fish, or etc.)?” The intake level included eight categories: “hardly eat,” “1 to 3 times per month,” “1 to 2 times per week,” “3 to 4 times per week,” “5 to 6 times per a week,” “once per day”, “twice per day,” or “≥ 3 times per day.” We converted the category into a continuous variable that represented intake frequency per week, i.e., “hardly eat” was coded as 0.0, “1 to 3 times per month” as 0.46, “1 to 2 times per week” as 1.5, “3 to 4 times per week” as 3.5, “5 to 6 times per week” as 5.5, “once per day” as 7.0, “twice per day” as 14.0, and “≥ 3 times per day” as 21.0. Considering that a year has 365.25 days, the intake frequency for the answer “1 to 3 times per month” was calculated as $\frac{1+3}{2}\times 12\div 365.25\times 7=0.46$ times per week.

### Adjustment variables

In the present study, the association analysis was adjusted for age and sex. In some analyses, study region, alcohol consumption, and/or alcohol drinking frequency were additionally used for the adjustment. Age, sex, and study region were obtained from the questionnaire. Alcohol drinking frequency was defined according to the question, “How frequently do you drink alcohol?” with seven categories—“hardly drink” (coded as 0.0), “less than 1 times per month” (coded as 0.12), “1 to 3 times per month” (coded as 0.46), “1 to 2 times per week” (coded as 1.5), “3 to 4 times per week” (coded as 3.5), “5 to 6 times per week” (coded as 5.5), and “everyday” (coded as 7.0). Alcohol consumption was calculated by summing the amount of alcohol in grams per day obtained from beer, red wine, white wine, highballs/cocktails, rice wine, and distilled spirits.

### DNA sampling, genotyping, and quality control

The Oragene DNA (OG-500) Collection Kit (DNA Genotek Inc., Ottawa, Ontario, Canada) was used for the collection, stabilization, and transportation of saliva samples. Genomic DNA was extracted from saliva by genotyping technology according to the manufacturer’s instructions. The participants were genotyped using two platforms: the HumanCore-12 + Custom BeadChip (Illumina Inc., San Diego, CA, USA) containing 302,072 markers and the HumanCore-24 + Custom BeadChip (Illumina) containing 309,725 markers. In this study, we used 296,675 markers included in both genotyping platforms.

After excluding subjects who did not live in Japan, 12,603 participants remained. According to a previous study [5], we divided our study participants into eight regional groups: “Hokkaido,” “Tohoku,” “Kanto-Koshinetsu,” “Tokai-Hokuriku,” “Kinki,” “Chugoku-Shikoku,” “Kyushu,” and “Okinawa.” We then applied the quality control and association analysis procedures described below for each regional group.

Single nucleotide polymorphism (SNP) markers with low call rates (< 0.95), low Hardy–Weinberg equilibrium exact test *P* values (< 1 × 10^−6^), or low minor allele frequencies (MAFs; < 0.01) were filtered out. Subjects who had inconsistent sex information between genotype and questionnaire, who had a low call rate (< 0.95), or who had an estimated non-Japanese ancestry [5, 17] were excluded. In addition, we excluded either close relationship pairs determined by the identity-by-descent method (PI_HAT > 0.1875) as in previous studies [8, 18, 19]. These quality control procedures were performed using PLINK [20, 21] (version 1.90b3.42) and Eigensoft [17] (version 6.1.3) software.

### Genome-wide association and meta-analysis

For each regional group, the association between each variant and fish intake frequency was tested by a linear regression model with adjustment for age, sex, and population stratification (5 principal components). Inflation of test statistics due to confounding from population stratification was assessed by calculating the inflation factor (*λ*), which is defined as the median of the observed test statistic divided by the median of the expected test statistic [17]. An inflation factor near 1.0 (i.e., 0.95–1.05) indicates that confounding from population stratification has been well adjusted for. This genome-wide association analysis was performed using PLINK software.

Summary statistics (beta coefficients and their standard error) from eight regional groups were meta-analyzed using a fixed-effect model and the inverse-variance weighting method with METAL software [22] (version 2011-03-25). Variants with a meta-analysis *P* value < 5 × 10^−8^ and < 1 × 10^−5^ were considered as GWS and having suggestive significance, respectively. Except for the 12q24 locus, variants achieving suggestive significance within 500 kb were grouped into a single locus, and for each locus, a lead variant was defined as the variant with the lowest *P* value in that locus. Regarding the 12q24 locus, variants within 2000 kb were considered as a single locus because the locus had a long-range disequilibrium. We checked the LD (*R*^2^) in the Japanese population between the lead and other variants using LDlink [6]. We confirmed that significant variants (*P* > 1 × 10^−5^) were in moderate or high LD (*R*^2^ > 0.3) with the lead variant for each locus.

## Additional files

Additional file 1: **Figure S1.** Male vs. female subgroup analysis with adjustment for alcohol consumption. **Figure S2.** Male vs. female subgroup analysis with adjustment for alcohol drinking frequency. **Figure S3.** Younger vs. older subgroup analysis with adjustment for alcohol consumption. **Figure S4.** Younger vs. older subgroup analysis with adjustment for alcohol drinking frequency. **Table S1.** Association between rs11066015 and fish intake frequency according to study regions. **Table S2.** Lead variants associated with fish intake frequency with adjustment for age, sex, population stratification, and body mass index. **Table S3.** Association between rs11066015 and fish intake frequency with and without adjustment for alcohol drinking. **Table S4.** Characteristics of the study participants stratified by sex. **Table S5.** Characteristics of the study participants stratified by age. **Table S6.** Correlation between fish intake and alcohol drinking frequencies. (DOCX 290 kb)

## Data Availability

All data analyzed during this study are included in this published article and its additional files. Other data are available from the authors upon reasonable request.

## Abbreviations

ALDH2: Aldehyde dehydrogenase 2 family member
BMI: Body mass index
DHA: Docosahexaenoic acid
EPA: Eicosapentaenoic acid
GWAS: Genome-wide association study
MAF: Minor allele frequencies
SNP: Single nucleotide polymorphisms
