# Supplementary material for: Identification of the 12q24 locus associated with fish intake frequency by genome-wide meta-analysis in Japanese populations
Source: Genes Nutr. 2019 Jul 5;14:21. doi: 10.1186/s12263-019-0646-6 (PMC6612078; doi:10.1186/s12263-019-0646-6)
Supplement: Supplementary file 1 — Figure S1. Male vs. female subgroup analysis with adjustment for alcohol consumption. Figure S2. Male vs. female subgroup analysis with adjustment for alcohol drinking frequency. Figure S3. Younger vs. older subgroup analysis with adjustment for alcohol consumption. Figure S4. Younger vs. older subgroup analysis with adjustment for alcohol drinking frequency. Table S1. Association between rs11066015 and fish intake frequency according to study regions. Table S2. Lead variants associated with fish intake frequency with adjustment for age, sex, population stratification, and body mass index. Table S3. Association between rs11066015 and fish intake frequency with and without adjustment for alcohol drinking. Table S4. Characteristics of the study participants stratified by sex. Table S5. Characteristics of the study participants stratified by age. Table S6. Correlation between fish intake and alcohol drinking frequencies. (DOCX 290 kb) [file 12263_2019_646_MOESM1_ESM.docx]

**Supplementary information for ‘Identification of the 12q24 locus associated with fish intake frequency by genome-wide meta-analysis in Japanese populations’**

Maki Igarashi, Shun Nogawa, Kaoru Kawafune, Tsuyoshi Hachiya, Shoko Takahashi, Huijuan Jia, Kenji Saito, Hisanori Kato

Supplementary Figures S1 to S4

Supplementary Tables S1 to S6

**
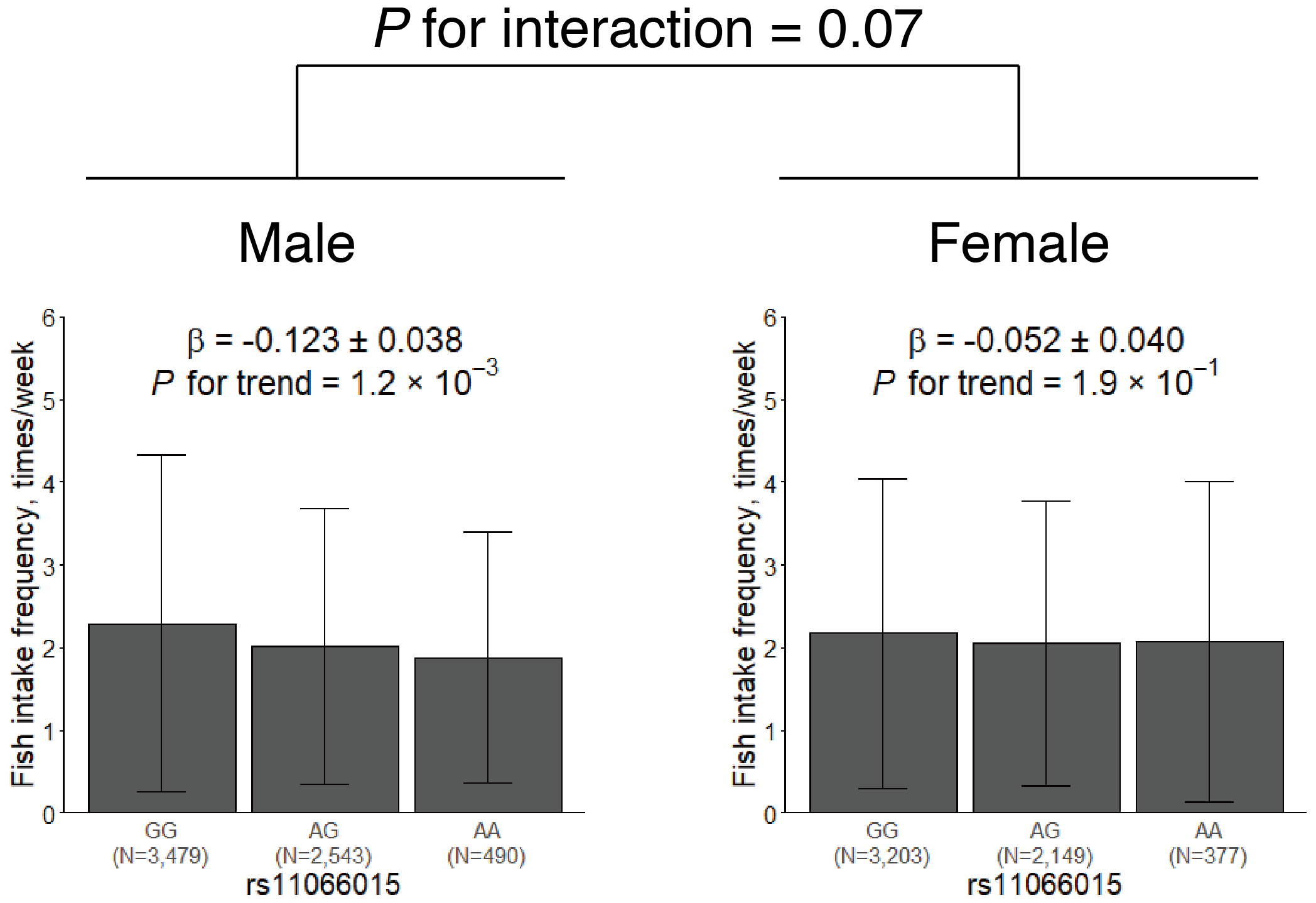
**

**Figure S1. Male vs. female subgroup analysis with adjustment for alcohol consumption.** The *x*-axis represents genotype of the lead variant at 12q24 (rs11066015) –– *i.e.*, GG, AG, or AA –– and the *y*-axis represents fish intake frequency in times per week.

**
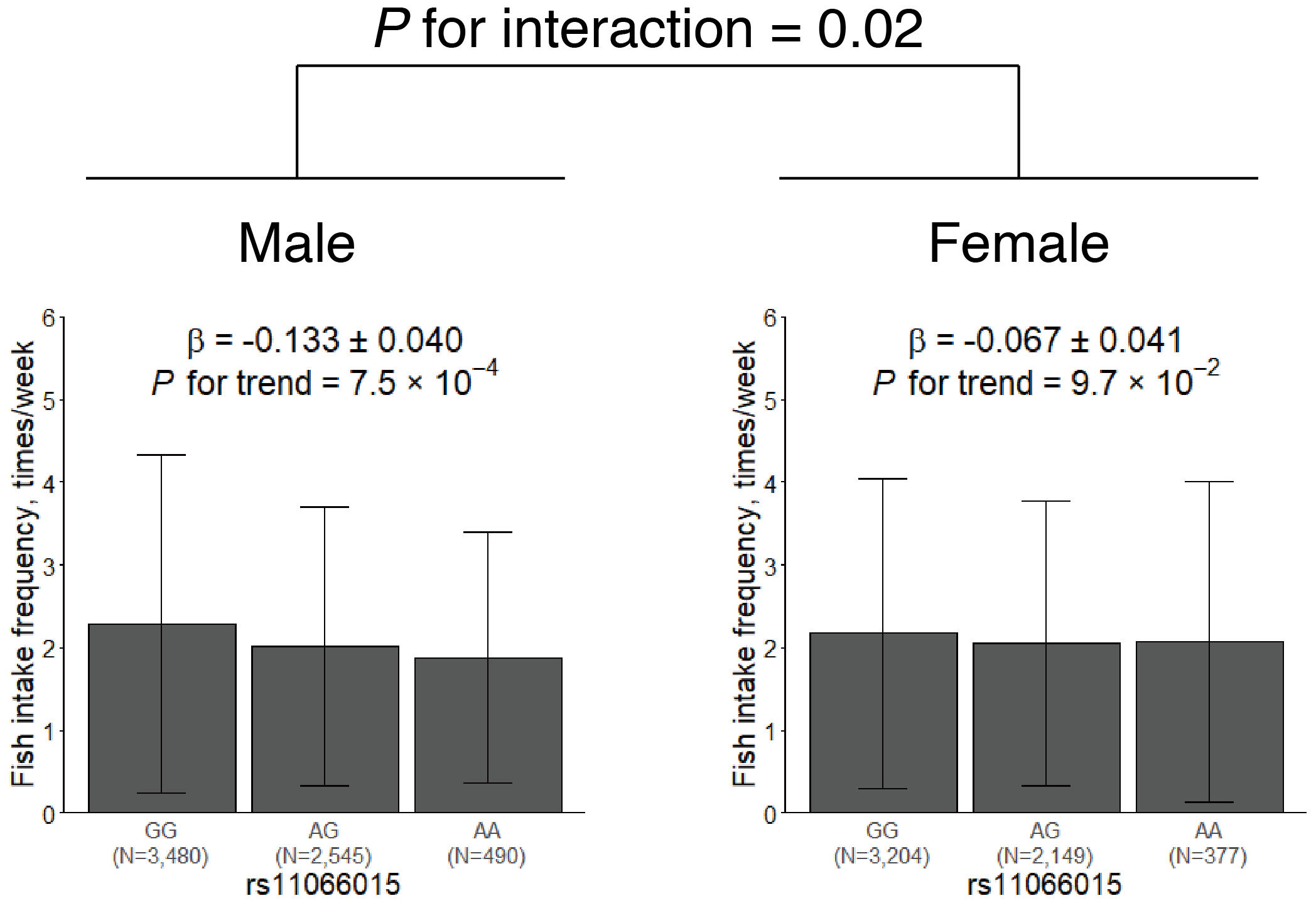
**

**Figure S2. Male vs. female subgroup analysis with adjustment for alcohol drinking frequency.** The *x*-axis represents genotype of the lead variant at 12q24 (rs11066015) –– *i.e.*, GG, AG, or AA –– and the *y*-axis represents fish intake frequency in times per week.

**
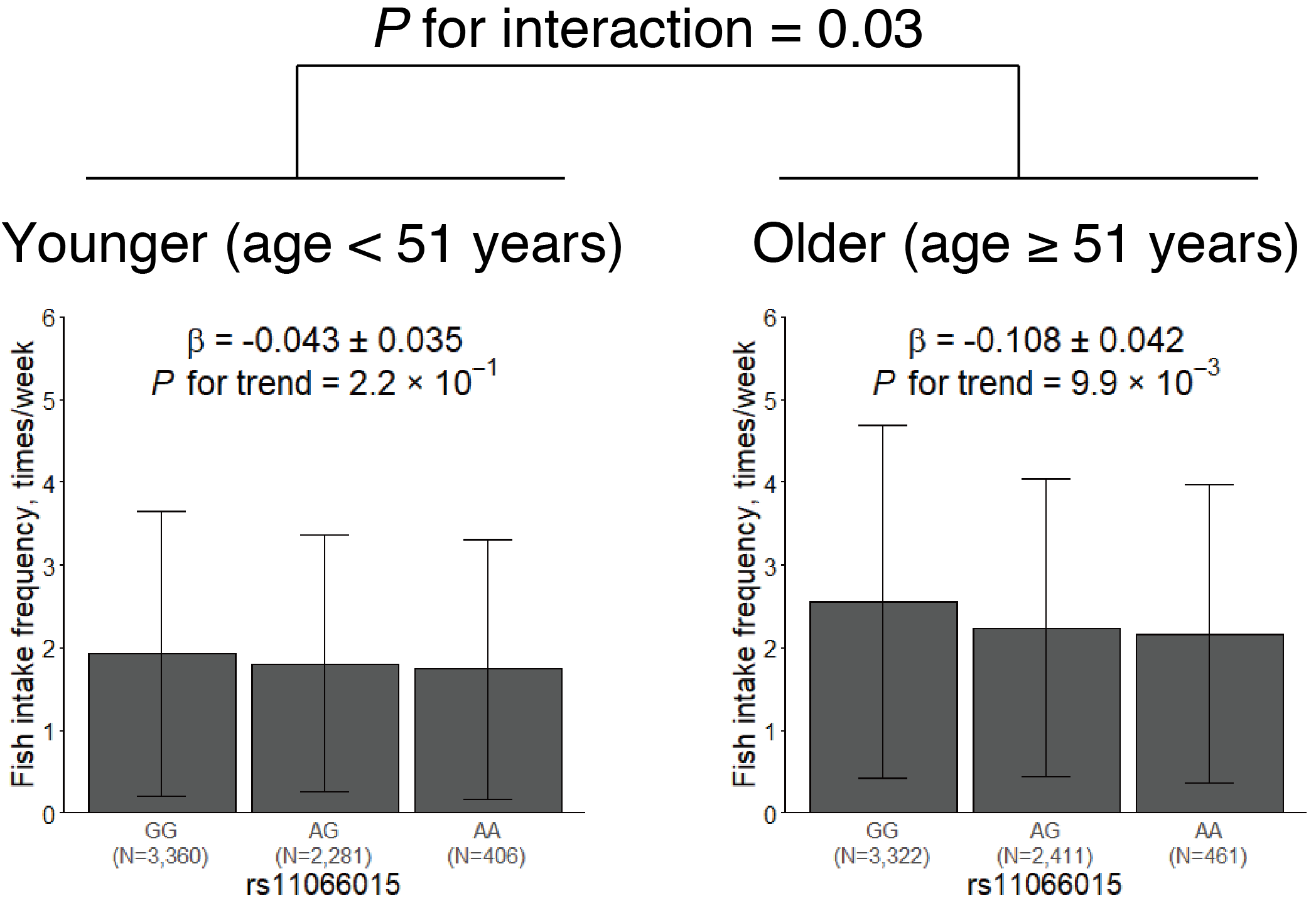
**

**Figure S3. Younger vs. older subgroup analysis with adjustment for alcohol consumption.** The *x*-axis represents genotype of the lead variant at 12q24 (rs11066015) –– *i.e.*, GG, AG, or AA –– and the *y*-axis represents fish intake frequency in times per week.

**
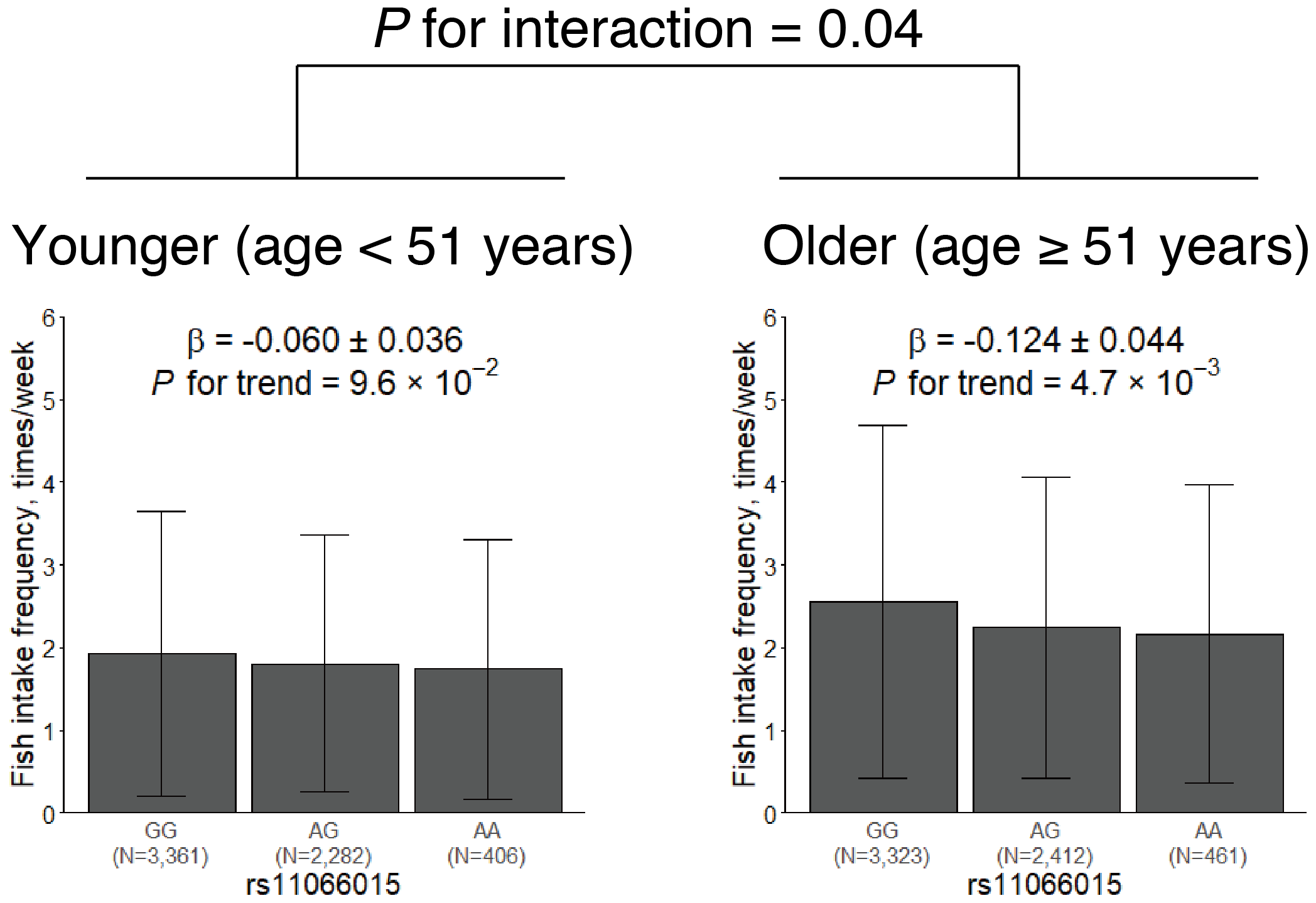
**

**Figure S4. Younger vs. older subgroup analysis with adjustment for alcohol drinking frequency.** The *x*-axis represents genotype of the lead variant at 12q24 (rs11066015) –– *i.e.*, GG, AG, or AA –– and the *y*-axis represents fish intake frequency in times per week.

**Table S1. Association between rs11066015 and fish intake frequency according to study regions**

| Study region | EAF | β | SE(β) | *P* |
| --- | --- | --- | --- | --- |
| Hokkaido | 0.215 | -0.094 | 0.148 | 0.52 |
| Tohoku | 0.194 | -0.298 | 0.119 | 0.01 |
| Kanto-Koshinetsu | 0.257 | -0.127 | 0.042 | 0.003 |
| Tokai | 0.291 | -0.282 | 0.076 | 0.0002 |
| Kinki | 0.296 | -0.200 | 0.059 | 0.001 |
| Chugoku-Shikoku | 0.265 | -0.134 | 0.085 | 0.12 |
| Kyushu | 0.259 | -0.167 | 0.083 | 0.05 |
| Okinawa | 0.186 | -0.240 | 0.287 | 0.41 |
| Meta-analysis | 0.265 | -0.174 | 0.027 | 5.4 × 10^-11^ |

EAF, effect allele frequency; SE, standard error

**Table S2. Lead variants associated with fish intake frequency with adjustment for age, sex, population stratification, and body-mass index**

| SNP | Chr | Position | Gene(s) | EA | NEA | EAF | Beta | SE(Beta) | *P*_association_ | *I*^2^ | *P*_heterogeneity_ |
| --- | --- | --- | --- | --- | --- | --- | --- | --- | --- | --- | --- |
| rs11758482 | 6 | 2,438,231 | *GMDS–C6orf195* | G | A | 0.404 | 0.107 | 0.024 | 7.2 × 10^-6^ | 47.5 | 0.06 |
| rs12003047 | 9 | 25,527,621 | *IZUMO3–TUSC1* | C | T | 0.015 | 0.440 | 0.097 | 6.1 × 10^-6^ | 53.6 | 0.03 |
| rs11066015 | 12 | 112,168,009 | *ACAD10* | A | G | 0.265 | -0.175 | 0.027 | 4.2 × 10^-11^ | 0.0 | 0.63 |
| rs1201914 | 20 | 59,274,641 | *CDH26–CDH4* | G | A | 0.452 | -0.117 | 0.023 | 5.0 × 10^-7^ | 0.0 | 0.55 |

SNP, single nucleotide polymorphism; Chr, chromosome; EA, effect allele; NEA, non-effect allele; EAF, effect allele frequency; SE, standard error

Chromosomal positions are according to the human genome assembly version GRCh37/hg19.

**Table S3. Association between rs11066015 and fish intake frequency with and without adjustment for alcohol drinking**

| Adjustment | β | SE(β) | *P* |
| --- | --- | --- | --- |
|  |  |  |  |
| Age, sex, study region | -0.174 | 0.026 | 4.3 × 10^-11^ |
| Age, sex, study region, alcohol consumption (g/day) | -0.089 | 0.028 | 1.2 × 10^-3^ |
| Age, sex, study region, alcohol drinking frequency (times/week) | -0.104 | 0.028 | 2.5 × 10^-4^ |

**Table S4. Characteristics of the study participants stratified by sex**

|  | Male | Female | *P* |
| --- | --- | --- | --- |
| *N* | 6,516 | 5,731 | - |
| Female, % | 0.0 | 100.0 | - |
| Age, year (mean ± SD) | 50.6 ± 13.5 | 49.9 ± 12.7 | 0.001 |
| BMI, kg/m^2^ (mean ± SD) | 23.9 ± 3.4 | 22.1 ± 3.8 | <0.001 |
| Fish consumption, times/week (mean ± SD) | 2.15 ± 1.88 | 2.12 ± 1.82 | 0.36 |
| Current alcohol drinkers, % | 70.2 | 51.9 | <0.001 |
| Current alcohol consumption, g/day (mean ± SD)* | 13.7 ± 14.6 | 8.3 ± 10.7 | <0.001 |

*Among current drinkers

BMI, body-mass index; SD, standard deviation

*P*-values were calculated using χ^2^ test for sex and current alcohol drinkers or Student *t*-test for other variables

**Table S5. Characteristics of the study participants stratified by age**

|  | Younger  (age < 51 years) | Older  (age ≥ 51 years) | *P* |
| --- | --- | --- | --- |
| *N* | 6,050 | 6,197 | - |
| Female, % | 46.5 | 47.1 | 0.48 |
| Age, year (mean ± SD) | 39.0 ± 7.5 | 61.3 ± 6.5 | - |
| BMI, kg/m^2^ (mean ± SD) | 22.8 ± 3.9 | 23.3 ± 3.5 | <0.001 |
| Fish consumption, times/week (mean ± SD) | 1.87 ± 1.65 | 2.40 ± 2.00 | <0.001 |
| Current alcohol drinkers, % | 61.8 | 61.4 | 0.67 |
| Current alcohol consumption, g/day (mean ± SD)* | 9.8 ± 12.5 | 13.3 ± 14.1 | <0.001 |

*Among current drinkers

BMI, body-mass index; SD, standard deviation

*P*-values were calculated using χ^2^ test for sex and current alcohol drinkers or Student *t*-test for other variables

**Table S6. Correlation between fish intake and alcohol drinking frequencies**

| Group | Pearson correlation coefficient | Spearman correlation coefficient |
| --- | --- | --- |
| All | 0.108 | 0.104 |
| Males | 0.144 | 0.150 |
| Females | 0.061 | 0.053 |
| Younger (age < 51 years) | 0.075 | 0.083 |
| Older (age ≥ 51 years) | 0.097 | 0.104 |
| Younger males | 0.074 | 0.099 |
| Younger females | 0.068 | 0.047 |
| Older males | 0.156 | 0.160 |
| Older females | 0.035 | 0.060 |

Correlation coefficients between fish intake frequency and alcohol drinking frequency were shown.
